# Isolated Myeloperoxidase Immunohistochemical Expression in Bone Marrow Biopsy Depicts Clinical Outcomes in Adults with Typical B-Acute Lymphoblastic Leukemia

**DOI:** 10.31557/APJCP.2021.22.7.2143

**Published:** 2021-07

**Authors:** Maha Mohamed Fawzy, Amal Abd El hafez, Shaimaa EL-Ashwah, Tarek E. Abouzeid, Maryan Waheeb Fahmi, Maha Saif, Metwaly Ibrahim Mortada, Mayada A. Ghannam, Heba Sheta

**Affiliations:** 1 *Pathology Department, Faculty of Medicine, Mansoura University, Mansoura, Egypt. *; 2 *Clinical Hematology Unit, Oncology Center, Faculty of Medicine, Mansoura University, Mansoura, Egypt. *; 3 *Internal Medicine Department, Oncology Center, Faculty of Medicine, Mansoura University, Mansoura, Egypt. *; 4 *Hematology Unit, Clinical Pathology Department, Faculty of Medicine, Mansoura University, Mansoura, Egypt. *

**Keywords:** Immunohistochemistry, response to therapy, survival, flowcytometry, adult B-acute lymphoblastic leukemia

## Abstract

**Introduction:**

Recently, isolated myeloperoxidase expression (isoMPO) has been documented in B-acute lymphoblastic leukemia (B-ALL) and several contradictory studies addressed its clinical significance in pediatric patients.

**Aim:**

In this study, isoMPO was evaluated in bone marrow biopsies (BMB) from adults with B-ALL using immunohistochemistry (IHC) in relation to a number of risk-stratification factors and patients’ outcomes.

**Methods:**

Sixty B-ALL adult patients were selected upon electronic database search. Demographic, clinical, laboratory, therapy and survival data were reviewed and tabulated. Flowcytometry (FCM), histopathology and IHC available material were reviewed to confirm the diagnostic criteria according to our standard laboratory protocols. IHC was performed on BMB using antiMPO. Cases were divided into MPO+ve and MPO-ve based on a 3% blast cell staining threshold.

**Results:**

Using IHC, 26.7% of B-ALLs were MPO+ve, in most of which ≥10% of blasts were stained. Among standard risk-stratification factors, isoMPO was associated with a mean WBC count above 30x10^9^/L. MPO+ patients achieved therapeutic complete remission at lower rates and were more prone to progressive/refractory disease and relapse. There was a concordant expression of MPO in FCM and IHC. All of the aforementioned parameters reached the level of significance when compared to the MOP-ve group. Kaplan-Meier curves revealed a significantly lower survival probability for the MPO+ group than the MOP-ve one (p= 0.0066; Log-rank test) and also when separating MPO+ and -ve patients by gender (p= 0.0033; Log-rank test).

**Conclusion:**

isoMPO occur in a considerable percentage of B-ALL in adults contributing to misdiagnosis. It depicts poor outcomes and might be introduced as a B-ALL risk-stratification factor.

## Introduction

B-acute lymphoblastic leukemia (B-ALL) is a precursor B-cell lineage-derived neoplasm composed of small to medium-sized blasts. It is typically detected in bone marrow (BM) and peripheral blood of patients presenting with evidence and consequences of BM failure with frequent involvement of extra-medullary sites. Worldwide, the estimated annual incidence of ALL is 1-4.75 cases per 100,000 population with approximately 80-85% being of B-cell type. Although ALL is primarily a childhood disease and 75% of cases occur in the first 6 years of life, a second peak occurs in adults at the age of 50 years where B-cell lineage constitute 75% of cases in this age (Borowitz et al., 2017a; Terwilliger and Abdul-Hay, 2017). 

Despite the significant improvement in pediatric patients’ outcomes, B-ALL represents a devastating, poor-prognosis disease in adults as only 30-40% of those patients achieve long-term remission (Terwilliger and Abdul-Hay, 2017; Jordaens et al., 2020). In the era of personalized medicine, it may be important to identify explicit prognostic parameters that allow better patients’ stratification aiming to provide the optimum therapeutic regimen for each particular case. 

In addition to blast cell morphology, B-ALL is diagnosed by flow-cytometric (FCM) detection of B-cell markers CD19, CD79a, and CD22; in combination or at high intensity. Blasts are typically positive for terminal deoxynucleotidyl transferase (TdT; 91%), CD10 (CALLA), CD24 and PAX5, whereas CD20 and CD34 are variably expressed and CD45 may be absent or dim. For CD79a and TdT, a 10% expression threshold is considered positive. Noteworthy, CD79a and PAX5 are most frequently used to demonstrate B-cell differentiation in tissue sections. CD13 and CD33 myeloid/cross lineage antigens can be expressed and a subset of B-ALL may be Philadelphia chromosome-positive (Ph+), but the myeloid-specific CD117 should not be present and is used to differentiate between ALL and acute myeloid leukemias (AMLs) (Chiaretti et al., 2014; Gupta et al., 2021). 

Recently, mixed phenotype acute leukemia (MPAL) was introduced as a distinct entity accounting for 2-5% of leukemias. MPALs are composed of bi-phenotypic or bi/multilineage blast cells that may show a B-lymphoid/myeloid, T-lymphoid/myeloid, B/T-lymphoid, or B/T-lymphoid /myeloid phenotype (Charles and Boyer, 2017; Khan et al., 2018). FCM is the most reliable method when a diagnosis of MPAL entails demonstrating co-expression of lymphoid and myeloid differentiation antigens on the same cell, while immunohistochemistry (IHC) in tissue sections, or with enzyme cytochemistry (EC) on smears coupled with FCM are used in other cases that requires demonstration of two distinct, different-phenotype leukemic populations. To confirm the myeloid component of MPAL, myeloperoxidase (MPO) is the single most specific hallmark, but due to technical factors associated with MPO measurement, a diagnosis of MPAL necessitates additional criteria as: expression of myeloid antigens such as CD117, very bright CD13 and CD33, expression of monocytic markers or heterogeneity of antigen expression (Borowitz et al., 2017b). From experts’ opinion, safe MPO thresholds are 10-13% in FCM (using isotype control) and 3% in IHC or EC (Guy et al., 2013; van den Ancker et al., 2013; Charles and Boyer, 2017). 

Outstandingly, isolated MPO expression (isoMPO) can be detected, most commonly by IHC but also sometimes by FCM in a subset of typical B-ALL specially in adults (Borowitz et al., 2017a), without fulfilling the diagnostic criteria of MPAL. Some investigators considered MPO expression in B-ALL as a diagnostic confounder in IHC (Du et al., 2020), or as a false positive FCM finding (Savasan et al., 2018). Nevertheless, the prognostic relations of this phenomenon were recently studied in pediatric B-ALL, and the data were controversial suggesting an association with higher risk and relapsing disease in some cohorts (Oberley et al., 2017, Li et al., 2019), or better clinical outcomes in other cohorts (Raikar et al., 2018; McGinnis et al., 2020). To the best of our knowledge, a limited number of studies have investigated isoMPO in adult patients with B-ALL using IHC (Arber et al., 2001), therefore it may be of importance to address further studies in this field. This study was conducted to evaluate the frequency of isoMPO expression in BM trephine biopsies obtained from adult patients diagnosed with otherwise typical B-ALL, using IHC. MPO positive (MPO+ve) B-ALL and MPO-negative (MOP-ve) B-ALL are further compared as regards the clinical features, routine laboratory parameters, FCM immunophenotyping (IPT) results, Philadelphia chromosome cytogenetic abnormality status and clinical outcomes measured by response to therapy, occurrence of relapse and overall survival (OS). 

## Materials and Methods

This retrospective cohort study was conducted at the Pathology Laboratories of the Oncology Center Mansoura University (OCMU) including a total of 60 patients diagnosed with B-ALL from January 2015 to December 2018 at the same center. Ethical approval was obtained from the Institutional Research Board (IRB, code: R.18.12.361.R1). Informed consent was obtained from all participants or their relatives whenever appropriate. The study was conducted in accordance with the current revision of Helsinki Declaration in dealing with human research (http://www.wma.net/e/policy/17-c_e.html).

*Patients’ Selection Criteria*

After reviewing the electronic database of the histopathology laboratory for the diagnosis of B-ALL, adult patients (18 years or older) with confirmed positive B-ALL blast cell deposits in BM biopsy (using the complete IHC panel) were selected. Cases with available clinical, routine laboratory, flow-cytometric IPT, therapy and follow-up data were included in the study after ensuring the availability of paraffin-embedded BM tissue sufficient for further IHC analysis. Cases that fulfilled the WHO diagnostic criteria of MPAL, as described later (Borowitz et al., 2017b), were excluded.

*Data Collection*

Demographic and clinical data were collected from electronic patient’s registry system including: age, gender, presence of symptoms (bone aches, repeated infections, bleeding tendency, anemia, B-symptoms: fever, drenching night sweats and loss of more than 10% of body weight over 6 months), presence extramedullary disease (such as lymphadenopathy or splenomegaly), peripheral blood cell count, hemoglobin (HB) level, erythrocyte sedimentation rate for first hour (ESR 1^st^ h.), and serum uric acid, creatinine and lactate dehydrogenase (LDH) levels were retrieved and tabulated. Philadelphia chromosome status (evaluated by fluorescent in situ hybridization; FISH), peripheral blood and BM aspirate general FCM IPT (precursor B, mature B or T lineage) and specific marker expression, treatment protocol, type of response to therapy, relapse and survival time from initial diagnosis (in days) were recorded.

*Standard Laboratory Protocols*

Blood and BM smears were morphologically examined using Leishman stain. At least 200 leukocytes on blood smears and 500 nucleated cells on BM smears are to be counted, with the latter containing spicules (Döhner et al., 2010). 

For the bone Marrow (BM) test, hemato-morphological and IPT analysis were used as the basis for diagnosis. IPT evaluation has been done using the following mixture of monoclonal antibody (mAb)-panels for AL diagnosis and to identify lineage affiliation, stage of maturation, and further characterization of AL: Cells were stained with fluorescein-conjugated Sm mAb against CD19, CD20, CD10, CD2, CD5, CD7, CD4, CD8, CD13, CD33, CD117, CD 64, CD14, CD36, CD33, CD34, and HLA-DR and intracellular mAb as cytoplasmic (cyto) CD79a, cytoCD3, MPO, and TdT. Samples on a BD FACS Canto™ flow-cytometer was analyzed. All mAbs were purchased from BD Pharmingen, San Diego, CA, USA. Acquisition and study of flow-cytometric data was conducted by independent analysis of results by two expert flow-cytometrists blinded to outcome data using the FACS DIVA software (BD, USA).

**Figure 1:**
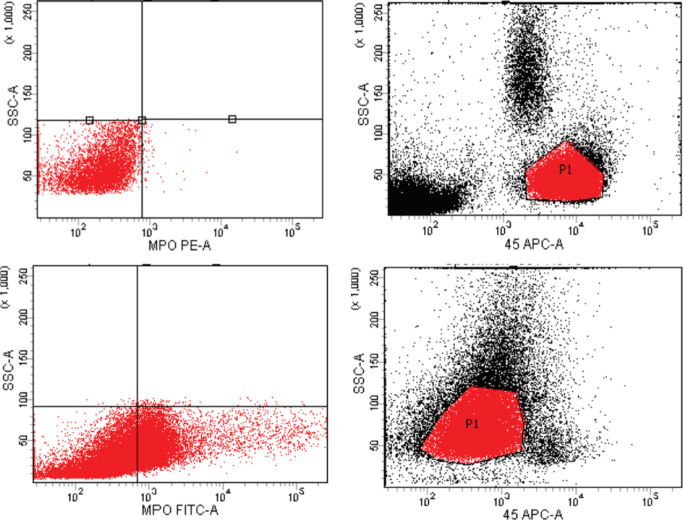
Positive and Negative Expression of Myeloperoxidase (MPO) in B-cell Acute Lymphoblastic Leukemia (B-ALL) Cases after Gating of Blast Cells Using CD45 gating Strategies Using Flowcytometry (FCM).

Based on CD45 dim expression and side-scatter properties, the blast gate was defined and calculated as a percentage of total gated events. Positive expression was evaluated as percentage and regarded when > 20% of blast cells were stained above the negative control (cutoff ≥ 20% of gated cells), while intracellular markers (e.g., MPO) were positive if more than 10% of blast cells expressed one of them (Bene et al., 1995; Bene et al., 2011; Ahuja et al., 2018) (Figure 1).

For histopathology, trephine BM biopsy specimens-fixed in 4% formalin solution, decalcified using S/P Decal Solution (Stephen’s Scientific, Riverdale, NJ) were processed into 3-4µm paraffin-serial sections stained with H&E. Biopsies suspicious for leukemic deposits were stained with the IHC antibodies directed against: CD34, CD117, CD99, CD3, CD20, CD19, CD79a, PAX5 (if available) and TdT. IHC was performed with Autostainer Link 48, using its optimized reagents and antibodies with pharmDx kits EnVisionTM FLEX Visualization Systems (Link code K8000) and EnVision FLEX Hematoxylin (Link code K8008) according to the user’s-guide standardized procedure pre-programmed into the autostainer software. Pre-treatment (dewaxing and dehydration) of FFPE sections with heat-induced epitope retrieval (HIER) using the 3-in-1 specimen preparation procedure was done with the following parameters: pre-heat temperature: 65°C; epitope retrieval: 97°C for 20 minutes; cool down to 65°C. The automated protocol is based on an indirect biotin-avidin system and uses a universal biotinylated immunoglobulin secondary antibody and diaminobenzidine (DAB) substrate. 

*Review of H&E and IHC-stained BM Sections*

All H&E and IHC-stained sections were reviewed by two pathologists using light microscope to evaluate specimen adequacy; overall cellularity; presence and percentage of normal BM series; presence and percentage of abnormal leukemic deposits and morphology of the infiltrate. Cell lineage (B/T/Myeloid) and maturity were determined by interpreting IHC positive markers and percentage of positivity.

*Diagnostic and Definition Criteria*

The diagnosis of B-ALL was based on interpretation of blast morphology, FCM data in peripheral blood and BM aspirate, in combination with H&E and IHC of BM trephine biopsy. 

*Response to therapy*

ALL patients were treated with pediatric-inspired protocol plus tyrosine kinase inhibitors (only for Philadelphia chromosome positive cases) for young/adolescent adult and Hyper-CVAD for adult patients. Relapsed/refractory cases were treated by either HAM (high dose cytarabine and mitoxantrone) or FLAG (fludarabine, cytarabine and G-CSF) protocol. Response was defined as complete response (CR) no circulating blasts or extramedullary disease, trilineage hematopoiesis (TLH) and < 5% blasts, absolute neutrophil count (ANC) > 1000/mcL, platelets > 100,000/mcL and no recurrence for 4 weeks. Refractory disease (RD) was failure to achieve CR at the end of induction. Progressive disease (PD) was defined as increase of at least 25% in the absolute number of circulating or bone marrow blasts or development of extramedullary disease. Relapsed disease was defined as reappearance of blasts in the blood or bone marrow (> 5%) or in any extramedullary site after a CR. Induction death (ID) was defined as death that occurred during induction and was unrelated to refractory leukemia (Alvarnas et al., 2012).

*Cell Morphology*

B-ALL blasts are relatively homogenous, with high nucleocytoplasmic ratio. The cells vary from small (scant cytoplasm, condensed nuclear chromatin, and indistinct nucleoli) to large (moderate amounts of light-blue cytoplasm, dispersed nuclear chromatin, and multiple variably prominent nucleoli). The nuclei are round or convoluted. Coarse azurophilic granules or cytoplasmic pseudopods (hand-mirror cells) are sometimes present. In BM biopsies, blasts are relatively uniform, with round to oval, indented, or convoluted nuclei, inconspicuous to prominent nucleoli and finely dispersed chromatin with variable mitotic Figures (Chiaretti et al., 2014; Borowitz et al., 2017a).

*MPAL Exclusion Criteria*

Cases with the following criteria (1) Positive CD117 combined with MPO or other myeloid or monocytic markers (CD11c, CD14, CD36, CD64, or lysozyme), (2) intense CD13 or CD33 (3) expression of at least 2 monocytic markers either intense or combined in absence of MPO, (4) intense cytoplasmic or surface CD3 or (5) heterogeneity of antigen expression (populations that are relatively bright for lymphoid markers show lower-level expression of myeloid antigens, and vice versa) (Arber et al., 2017; Borowitz et al., 2017a; Oberley et al., 2017; Li et al., 2019), were considered as MPAL B/myeloid, and thus excluded from the study.

*Myeloperoxidase IHC Staining and Evaluation*

About 4-um thickness paraffin-embedded sections on silanized glass slides were stained manually with rabbit polyclonal anti-human anti-Myeloperoxidase antibody (ab45977; abcam corporation, USA, diluted at 1/50, incubated for 1 hour) using heat-mediated antigen retrieval in citrate buffer pH 6.0 and blocking (5 minutes/peroxidase block and 10 minutes/protein block). Horseradish Peroxidase technique was applied. DAB was applied as a chromogen and hematoxylin for counterstaining as per provided antibody data sheet instructions. Appropriate negative control was performed for each staining run. Two pathologists, blinded to the clinical data, microscopically examined the MPO-stained slides. A threshold of at least 3% cytoplasmic MPO expression by the blasts was considered positive provided that the intensity is at least the same as the residual myeloid series positive internal control (Ahuja et al., 2018; Savasan et al., 2018). 

*Statistical Analysis*

Analysis was carried out with IBM Corp. SPSS (International Business Machines Corporation Statistical Product and Service Solutions), Version 21.0. Armonk, NY: IBM Corp., Chicago, USA. Data normality was tested with one-sample Kolmogorov-Smirnov test. Qualitative data were expressed as numbers and percentages, while continuous variables were presented as min-max and mean ± SD (standard deviation). Comparisons between MPO+ve and MPO-ve groups was tested using standard-t-test (means) and Chi-square *χ*^2^ test, while Fisher’s exact and Mann–Whitney tests were applied whenever appropriate. Kaplan-Meier test was used for survival analysis and statistical significance of differences among curves was determined by Log-Rank test. P-value was considered significant if ≤0.05. The smaller the p-value, the more significant were the results. 

## Results

*Study Group Criteria*

The study included 25 female and 35 male adult patients diagnosed with B-ALL during the study period. Patients ranged in age from 18-63 years and had an overall survival range from 9 to 4,834 days from initial diagnosis to the last follow-up. 

*Frequency of MPO Expression in IHC*

**Figure 2:**
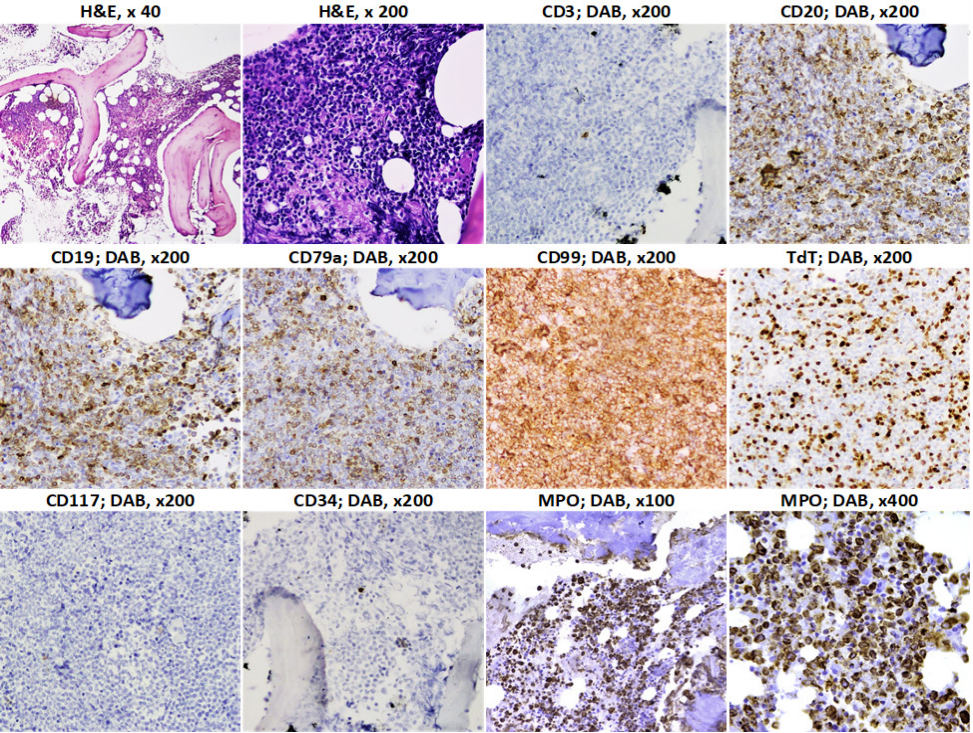
Hematoxylin and Eosin (H&E) and Immunohistochemical Features (IHC) of a Bone Marrow Biopsy in a Case of isoMPO Positive B-cell Acute Lymphoblastic Leukemia (B-ALL), Showing Blast Cell Deposits at Low and High-Power Magnifications. Blasts are Immunoreactive for CD20, CD19, CD 79a, CD99 (all membranous) and TdT (nuclear), but Unreactive for CD3, CD34 and CD117. Cytoplasmic Myeloperoxidase (MPO) Highlights the Immunoreactive B-cell Blasts in a Diffuse Pattern. DAB; Diaminobenzidine

Based on the IHC expression of MPO in the blast cell population of the corresponding BM biopsies, B-ALL patients were classified into two groups: an MPO+ve group comprised of 16 patients (26.7%) and an MPO-ve group including 44 patients (73.3%). Among the MPO+ biopsies, 6 (37.5%) showed ≥3-10% positivity of blast cells and the remainder (10 biopsies; 62.5%) showed ≥10% to diffuse positivity (Figure 2).

*Comparison of MPO-positive and MPO-negative B-ALL*

As demonstrated in table 1, MPO+ve patients tended to have higher mean age, total white blood cell (WBCs), neutrophil and platelet counts, and higher first hour erythrocyte sedimentation rate (ESR1^st^), and mean serum creatinine and uric acid levels. Whilst, MPO-ve group exhibited higher mean hemoglobin concentration and lactate dehydrogenase (LDH) levels and had an overtly higher mean OS time (741 days versus 431days for MPO +ve group). There was a minor difference in the means of blast cell count among the MPO+ve and MPO-ve groups (0.840625 and 0.8425581 x10^9^/L respectively). Yet, none of the above listed variables attained a statistically significant difference between the study groups (all p-values >0.05). 

Regarding the clinical features, most of the MPO+ve patients (87.5%) had B-symptoms, a half of them had associated lymphadenopathy and about 62.5% had splenomegaly at time of presentation. Though a lower percentage of MPO-ve patients presented with B-symptoms (61.3%), higher percentages revealed lymphadenopathy and splenomegaly (59 and 70.4% respectively). However, no statistically significant differences were found when both groups were compared in respect to these features. There was a significant statistical difference in the therapeutic response (p=0.0017), as above 70% of MPO-ve patients achieved a complete response (CR), compared to 43.8% of the MPO+ve patients who had a higher tendency for progressive disease (PD; 6.3 vs 0%) or refractory diseases (RD; 12.5 vs 2.2%). However, 20.5% of MPO-ve patients were candidates for therapy-related induction death (ID). During their follow-up period, 75% of MPO+ve patients suffered relapse compared to a percentage of 18.2% among the MPO-ve group, and this difference created a statistically significant p-value (p=0.0009). 

Using FCM, MPO positivity was detected in 18.3% of all B-ALL cases being more frequent in IHC MPO+ve cases (56.3%) and less frequent in the IHC MPO-ve cases (10%) with a significant statistical difference between both groups. Non-intense CD13 and CD33 expression was observed in 16.7% of B-ALL cases including 12.5 and 18.7% of MOP+ve cases, and 18.2 and 15.9% of MOP-ve cases respectively. CD117 was positive in 2 out of 44 MOP-ve otherwise typical B-ALLs, while all MOP+ve cases were negative for CD177 by FCM. However, none of these findings was of statistical significance. FISH analysis revealed t (9;22) in about 13.6% of MPO-ve B-ALLs, however, none of the MPO+ve patients had a similar translocation pattern. For the Philadelphia chromosome status, a significant statistical difference was observed between both groups (p=0.027; Table 2).

*Survival analysis*

**Figure 3:**
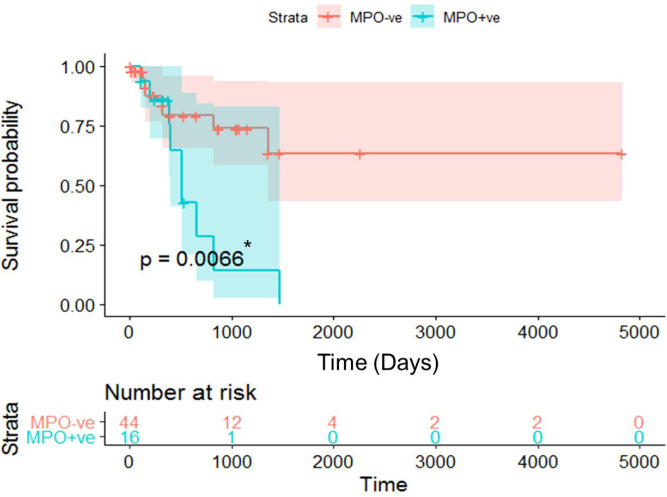
Kaplan-Meier Plot of Overall Survival (OS) from Date of Diagnosis in Patients with Myeloperoxidase (MPO)–positive (+ve) and MPO–negative (-ve) B-acute Lymphoblastic Leukemia. MPO+ve Patients have a Significantly Lower OS Probability; p= 0.0066 (Log-rank test). Cross Marks Represent Censored Data, *p-value is Significant if ≤0.05

**Figure 4:**
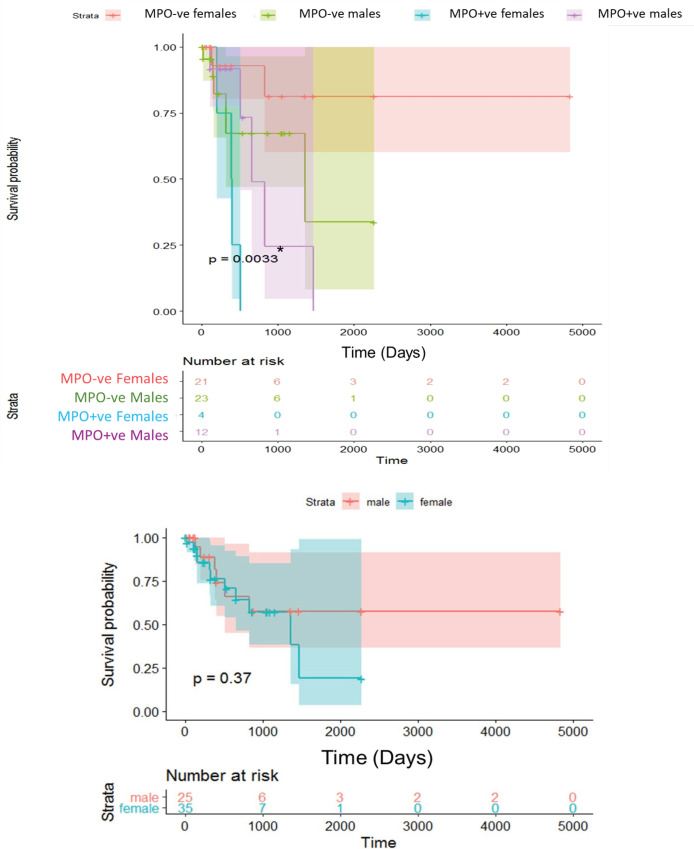
Kaplan-Meier Plot of Overall Survival (OS) from Date of Diagnosis in Patients with Myeloperoxidase (MPO)–positive (+ve) and MPO–negative (-ve) B-acute Lymphoblastic Leukemia (B-ALL). Female and Male MPO+ve Patients have Significantly Lower OS Probabilities; p= 0.0033 Compared to Gender Matching MPO-ve Patients (Log-rank test, upper panel). No Observed OS Probability Difference among the Included B-ALL Females and Males per se (p=0.37; Log-rank test, lower panel). Cross Marks Represent Censored Data, *p-value is significant if ≤0.05

As demonstrated in the Kaplan-Meier survival curves, MPO+ve B-ALL patients had significantly reduced OS probability compared to the MPO-ve patients (p =0.0066; Log-Rank test, Figure 3). Comparing survival probabilities in male and female patients disclosed that MPO+ve B-ALL male and female patients had lower survival probabilities compared to the same gender MPO-ve patients (p =0.0033; Log-Rank test), however, no significant difference was found when survival probability of B-ALL patients was compared to gender per se excluding the MOP status (p =0.37; Log-Rank test, Figure 4).

**Table 1:** Demographic and Laboratory Parameters Compared between MPO+ve and MPO-ve B‐ALL Cases

	B-ALL Group			
Variable	Total (Range)	IHC, MPO+ve (Mean±SD)	IHC, MPO-ve (Mean±SD)	P-value
Age (years)	18-63	37.375±16.87947	33.46512±15.54463	0.4478
WBCs (x10^9^/L)	1.2-162	36.725±48.42347	25.10977±33.51976	0.3672
Neutrophils (x10^9^/L)	0.09-34	3.759333±8.417538	2.925349±5.228171	0.7134
Blasts (x10^9^/L)	0.452644-1.228605	0.840625±0.1551545	0.8425581±0.1428468	0.9221
Hemoglobin gm/dl	5.6-12.9	7.9875± 1.560288	8.311628±1.93184	0.4747
Platelets (x10^9^/L)	5-317	67.65625±72.44463	59.77674±75.72634	0.6909
LDH (IU)	106.2-9547	899.1538±1021.195	1260.668±1727.76	0.2049
ESR 1^st^ hour	10-147	68.375±39.65544	51.72222±23.26018	0.2426
Serum creatinine (mg/dl)	0.6-6.1	2.25±2.133542	1.236512±0.8808542	0.08245
Serum uric acid (mg/dl)	2.8-36	12.03125±8.471735	7.730244±4.888676	0.08487
OS (days)	9 - 4834	431.125±349.1194	741.0698±1080.795	0.1158
Total No. (percent)	60 (100%)	16 (26.7%)	44 (73.3%)	

IHC, immunohistochemistry; MPO, myeloperoxidase; +ve, positive; -ve, negative; WBCs, white blood cell; ESR1^st^, first hour erythrocyte sedimentation rate; LDH, lactate dehydrogenase; OS, overall survival; No, number; * p-value is significant if ≤0.05.

**Table 2:** Clinicopathological Features, Therapeutic Responses, Aberrant Flowcytometry Findings and Philadelphia Chromosome Status Compared between MPO+ve and MPO-ve B‐ALL Cases

Variable	Total No (%)	IHC, MPO+ve; No (%)	IHC, MPO-ve; No (%)	X^2^	P-value
B-symptoms	41 (68.3)	14 (87.5)	27 (61.3)	2.5947	0.1072
Extramedullary disease					
Lymphadenopathy	34 (56.7)	8 (50)	26 (59)	0.1114	0.7385
Splenomegaly	41 (68.3)	10 (62.5)	31 (70.4)	0.0739	0.7857
Response to therapy					
CR (complete response)	38 (63.3)	7 (43.8)	31 (70.4)		
PR (partial response)	9 (15)	6 (37.5)	3 (6.8)	17.162	0.0017*
ID (induction death)	9 (15)	0(0)	9 (20.5)		
PD (progressive disease)	1 (1.7)	1(6.3)	0 (0)		
RD (refractory disease)	3 (5)	2(12.5)	1 (2.2)		
Relapse	20 (33.3)	12 (75)	8 (18.2)	13.9	0.0009*
Flowcytometry					
CD13	10 (16.7)	2 (12.5)	8 (18.2)	2.28	0.31
CD33	10 (16.7)	3 (18.7)	7 (15.9)	3.7	0.155
CD117	2 (3.3)	0 (0)	2 (4.5)	3.7	0.156
MPO	11(18.3)	9 (56.3%)	4 (10)	12.72	0.0003
t (9;22)	6 (10)	0 (0)	6 (13.6)	7.15	0.027*
Total No. (percent)	60 (100%)	16 (26.7%)	44 (37.3%)		

IHC, immunohistochemistry; MPO, myeloperoxidase; +ve, positive; -ve, negative; No, number; t, translocation; CD, cluster designation; FCM, Flow cytometry; *p-value is significant if ≤0.05.

## Discussion

MPO is a peroxidase enzyme encoded by a gene on human chromosome 17. It is contained within the primary granules of most granulocytes, starting from promyelocytes to the fully mature granulocytes. It is therefore, the hallmark of myeloid-lineage being detected in more than 80% of blasts in AML. Contrarily, lymphoblasts in ALL and mature lymphoid cells are MPO-negative (Omman and Kini, 2020). Though FCM, IHC and EC have been traditionally applied to detect MPO, a recent study demonstrated that IHC is the most sensitive technique specially in discrepant cases, depicting the superior advantage of IHC over FCM and its applicability in dry tap samples and in the set up where the facility of FCM is not available (Ahuja et al., 2018). Beside its diagnostic value, MPO status was used as a prognostic determinant for AML patients who may benefit from BM transplantation (Kim et al., 2012).

Although uncommon, MPO expression in ALL is known to occur. Nonetheless, its significance is not well-established. In fact, ALL blasts might express MPO -depending on the maturation of the progenitor cells- and trace MPO has been found in lymphoblasts at mRNA and protein levels (Serrano et al., 1999; Li et al., 2019). In the current study, isoMPO expression was detected in 26.7% of otherwise typical B-ALL marrow samples using IHC at a threshold of ≥3% and most of cases revealed a diffuse IHC expression in ≥10% of blasts, while 18.3% of FCM studied samples were positive, indicating a better detection-accuracy for IHC than FCM. Likewise, preceding studies reported a high frequency of MPO immunoreactivity in adult ALL of B- but not of T-cell lineage (17-23%) involving the majority of blasts in positive cases (Arber et al., 2001; Du et al., 2020), and negative or lower FCM results as well (Ahuja et al., 2018; Du et al., 2020). Reporting this finding seems of utmost importance to avoid misdiagnosis of ALL cases immunoreacting with MPO as AML or MPAL (Arber et al., 2001, Li et al., 2019), where isoMPO expression in IHC can be a confounder or a diagnostic challenger for lineage assignment in AL (Li et al., 2019; Du et al., 2020). Due to the discordant MPO expression results between IHC and FCM, lowering the MPO cutoff for FCM is suggested so as to increase its sensitivity (Ahuja et al., 2018).

In pediatric B-ALL population, isoMPO occur at a lower frequency than adults. Data coming from pediatric and young adults’ studies indicated discrepant criteria of isoMPO patients. In one study, MPO+ve patients tended to be older males without increased WBC counts or common B-ALL-related karyotypic findings, and showed an increased rate of relapse and a worse event-free survival than the patients with B-ALL who did not express MPO (Oberley et al., 2017). But another study that demonstrated a higher frequency of isoMPO expression, identified similar aberrant expression of myeloid markers and similar or even better clinical outcomes (as indicated by minimal residual disease) in the MPO+ve and -ve B-ALL, suggesting that these cases are not necessarily high-risk as a group and remain best classified as B-ALL rather than a separate entity (McGinnis et al., 2020).

In this cohort, 56.3% of IHC MPO+ve cases were also positive with FCM, indicating a considerable concordance between IHC and FCM methods for detection of MPO, and a better performance of IHC in this context. Nonetheless, 10% of IHC MPO-ve cases were positive with FCM. In the same vein, Ahuja et al. (2018), found discrepancies when compared MPO expression in AML using IHC, FCM, and cytochemistry in which IHC was positive in 22/28 of these cases, while FCM was positive in 14/28 cases. We agree with the previous study that it is important to employ more than one technique, and that IHC must be included for detection of MPO.

Comparing the standard risk-stratification factors in ALL (Hafiz et al., 2008; Terwilliger and Abdul-Hay, 2017), between the MPO+ve and MPO-ve cases, this work revealed no significant differences among MPO+ve and -ve patients in regard to age, B-symptoms, extramedullary disease, blood cell counts including the WBC, neutrophil and blast cell counts, HB, ESR, uric acid, creatinine and LDH mean values. Noteworthy, MPO+ve patients had a mean WBC count above the cutoff value of 30x10^9^/L, which has been considered as an independent prognostic factor for disease-free survival (DFS) and OS in B-ALL (Terwilliger and Abdul-Hay, 2017). Moreover, no significant differences were detected when comparing the aberrant FCM positivity of myeloid markers CD13, CD33 and CD117 in this work. We came across a single comparable study in which isoMPO was investigated in adult B-ALL patients (Arber et al., 2001). In the aforementioned study, no differences in age, sex distribution, peripheral blood count or CD33 expression were observed between the MPO+ve and MPO-ve groups, but the investigators reported extramedullary disease to be a common risk factor in the MPO-negative group and CD13 to be increased significantly in the MPO+ve group. However, our results are difficult to compare due to scarcity of studies in the same age group.

In adult ALL, the most common cytogenetic aberration with the greatest prognostic and therapeutic impacts is the Philadelphia chromosome, t(9;22) that occurs in 15-30% of adult ALL and associates with unfavorable criteria as older age and higher WBC counts. Whereas p210BCR-ABL is characteristic for chronic myeloid leukemia, a shorter version, p190BCR-ABL, predominates in Ph-positive ALL (Faderl et al., 2003). Among our patients, t(9;22) was detected 13.6% of MPO-ve B-ALL, but none of MPO+ve cases, and this was of statistical significance. On the contrary, t(9;22) was more frequent in the MPO+ve B-ALL in the study by Arber et al., (2001), yet, this finding did not reach statistical significance. This difference could be attributed to the existence of the so-called, Ph-like ALL, a subset of high-risk ALL without t(9;22) associated with poor response to induction chemotherapy and poor survival (Terwilliger and Abdul-Hay, 2017).

So far, the clinical prognostic value of MPO expression in AML has been controversial. It is logical that low MPO expression in AML patients should be associated with significantly lower rates of CR, DFS and OS than the high MPO expression (Kim et al, 2012; Dong et al., 2019). Despite this, an earlier study reported opposite results that a high proportion of MPO+ blasts before treatment establishes significantly unfavorable prognostic outcomes (Suic et al., 1992). This situation seems to apply also for the MPO status in B-ALL. Our patients in the MPO+ve group achieved CR at lower rates and were more prone to PD, RD and relapse, besides all of these parameters reached the level of significance when compared to the MOP-ve group. Furthermore, Kaplan-Meier survival curves, revealed a significantly lower survival probability for the former group than the latter one (p= 0.0066; Log-Rank test) and also when separating MPO+ and -ve patients by gender (p= 0.0033; Log-Rank test). Therefore, isoMPO might be suggested as a risk-stratification factor for B-ALL in adults. Yet, the same analysis in another study, suggested a trend toward significance in OS between MPO-ve and MPO+ve patients (P=.07), with a longer 5-year survival of MPO+ve adult patients compared with the age and gender-matching MPO-ve patients (Arber et al., 2001). To resolve this emerging disagreement, further studies are warranted to evaluate the clinical and outcome impacts of isoMPO expression in adults with B-ALL.

In conclusion, as detected by IHC on BM biopsies, isoMPO expression occur in a considerable percentage of otherwise typical B-ALL in adults, thus far contributing to misdiagnosis. MPO positivity is associated with a substantial number of unfavorable prognostic factors including a WBC count above 30x10^9^/L, lower CR rate, and higher PD, RD and relapsing disease rates as well as a lower OS survival probability, regardless patient’s gender. Although isoMPO is more frequently detected in BM samples using IHC than FCM, still there is a concordance the IHC and FCM findings. Further studies are recommended to verify the likelihood of introducing isoMPO as a risk-stratification factor for B-ALL in adults.

## Author Contribution Statement

All authors have equally participated according to specialty in: Conceptualization; Data curation; Formal analysis; Funding acquisition; Investigation; Methodology; Project administration; Resources; Software; Supervision; Validation; Visualization; Writing - original draft; Writing - review & editing.
